# Study Design and Participation Rates of the New York City Health and Nutrition Examination Survey, 2004

**Published:** 2006-06-15

**Authors:** Lorna E Thorpe, R Charon Gwynn, Jenna Mandel-Ricci, Sarah Roberts, Thomas R Frieden, Benjamin Tsoi, Lew Berman, Kathryn Porter, Yechiam Ostchega, Lester R Curtain, Jill Montaquila, Leyla Mohadjer

**Affiliations:** New York City Department of Health and Mental Hygiene; New York City Department of Health and Mental Hygiene, New York, NY; New York City Department of Health and Mental Hygiene, New York, NY; New York City Department of Health and Mental Hygiene, New York, NY; New York City Department of Health and Mental Hygiene, New York, NY; New York City Department of Health and Mental Hygiene, New York, NY, and Centers for Disease Control and Prevention, Epidemic Intelligence Service, Atlanta, Ga; Centers for Disease Control and Prevention, National Center for Health Statistics, Hyattsville, Md; Centers for Disease Control and Prevention, National Center for Health Statistics, Hyattsville, Md; Centers for Disease Control and Prevention, National Center for Health Statistics, Hyattsville, Md; Centers for Disease Control and Prevention, National Center for Health Statistics, Hyattsville, Md; Westat, Rockville, Md; Westat, Rockville, Md

## Abstract

**Introduction:**

Few state or local health agencies have accurate local-level information on the prevalence of the leading causes of morbidity and mortality. The New York City Health and Nutrition Examination Survey (NYC HANES) was designed as a new local surveillance initiative to determine the prevalence of health conditions among adult residents of New York City.

**Methods:**

Modeled after the National Health and Nutrition Examination Survey, the survey was initiated in June 2004 as a population-based cross-sectional study of New York City adults aged 20 and older. The survey was designed using a three-stage cluster sampling plan; 4026 households were randomly selected. Selected households were visited, and residents were given an initial eligibility screening questionnaire. Eligible participants were asked to schedule an appointment at an NYC-HANES–dedicated health center to complete the NYC HANES. A completed survey was defined as completion of a demographic interview and at least one examination component. Health conditions examined included cholesterol levels, diabetes status, blood pressure, environmental biomarkers, depression, anxiety, and antibodies to infectious diseases.

**Results:**

Of the 4026 households approached, eligibility screening questionnaires were completed for 3388 (84%) households, and 3047 survey participants were identified. Of the 3047 participants, 76% made an appointment, and 66% completed the survey. The overall response rate was 55% (n = 1999).

**Conclusion:**

NYC HANES is the first successful local-level examination survey modeled on NHANES. With periodic repetition, NYC HANES will provide surveillance information on leading causes of morbidity and mortality.

## Introduction

Public health surveillance is essential to monitor and control disease, yet few state or local health agencies have accurate information on the prevalence of the leading causes of morbidity and mortality, such as diabetes, hypertension, hypercholesterolemia, or depression. Self-reported information from telephone surveys can provide useful information about these conditions, but estimates are often inaccurate because of poor validity and reliability. Undiagnosed conditions are not reported, and accurate recall of conditions such as hypertension and hypercholesterolemia is poor (1-3).

Since the early 1960s, the U.S. National Center for Health Statistics has conducted the National Health and Nutrition Examination (NHANES) program as a series of surveys focusing on different population groups and health topics. In 1999, the program became a continuous program designed to collect data from a nationally representative sample of about 5000 individuals (4). For each of these surveys, participants undergo a detailed interview in their home followed by a physical examination in a survey-dedicated mobile examination center. Findings from NHANES have been used as the basis for public health interventions and health policies (5-7).

Although NHANES provides data on national public health problems in the United States, it does not provide state or local estimates of health problems, and no other similar infrastructure exists to collect objective local health data. In 2003, the New York City Department of Health and Mental Hygiene and the National Center for Health Statistics designed the first community-level version of the NHANES — the New York City HANES — to determine the prevalence and control of health status indicators among adults residing in New York City.

The NYC HANES was designed to provide prevalence information on 1) conditions that can be identified only through a physical examination (e.g., hypertension) or biologic specimen testing (e.g., diabetes) and 2) conditions that are not easily ascertained by a telephone survey (i.e., mental illness and conditions related to drug use, sexual behavior, incarceration, and domestic violence). Survey topics were selected to provide baseline data on priority chronic and infectious conditions for which intervention programs either exist or can be initiated (Table 1). The survey was designed so that periodic repetition (approximately every 5 to 7 years) will allow for population-based, outcome-oriented evaluation of local health interventions. Findings from the NYC HANES will complement but not duplicate information gathered from existing data sources within the New York City Department of Health and Mental Hygiene, including data from an annual random-digit–dialed telephone survey (8).

## Methods

### Sample design

NYC HANES was designed as a population-based, cross-sectional survey of adult residents of New York City, using a three-stage cluster sampling plan (Figure). In the first stage of the sample design, 144 segments were randomly selected as primary sampling units from a sampling frame of 21,169 segments across the city. The segments are based on counts of households from the 2000 U.S. census (9) and consist of a block or a group of proximal blocks within a given census tract; each segment has a required minimal total number of households. The sample of segments was selected with probability proportional to a measure of size.

**Figure:**
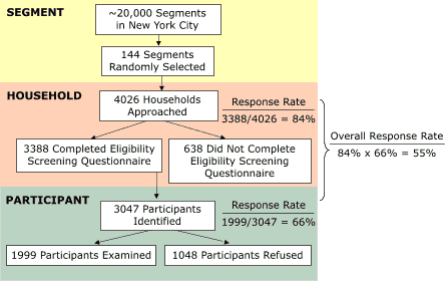
Sample design and participation rates of the New York City Health and Nutrition Examination Survey, 2004.

In the second stage of the sample design, a sampling frame of households was generated by sending field-staff teams to enumerate all dwelling units located in the 144 segments. A sample of 4026 households was randomly selected from the 144 segments.

In the third stage of the sample design, adults within households were selected for inclusion in the study. Eligible adults aged 20 and older were randomly selected based on an a priori computer-generated sampling flag. The sampling procedure was designed to select either zero, one, or two adults from each selected household, depending on the total number of adults residing in that unit. This design was chosen from among several considered to minimize variability in selection probabilities and maintain operational feasibility. Variation in selection probabilities was minimized by selecting fewer households in which an adult lived alone than households in which more than one adult lived. Operational efficiency was improved by ensuring that two adults would be selected from any household with three or more adults, thus reducing the number of households to be visited. With this design, a statistically efficient, approximately self-weighting sample of participants was attained, and the design effect due to intrahousehold correlation was minimized. Information from the 2000 U.S. census was used to obtain estimates for the number of one-person households, and the March 2001 Current Population Survey was used to obtain estimates for the number of two-, three-, four-, and five-or-more-person households (10). The parameters for the sample design are outlined in the Table 2.

### Sample size 

The target sample size for NYC HANES was 2000 adults; the sample size was selected to ensure statistical power comparable with the annual NHANES surveys conducted since 1999 (11). The study design team assumed that an initial eligibility screening questionnaire would be completed in 80% of the households targeted and that 75% of the eligible survey participants would complete the survey, yielding a final response rate of 60%.

### Study population 

#### Eligibility

The NYC HANES target population consisted of noninstitutionalized adult civilians aged 20 years or older residing in the five boroughs of New York City. This population included non-English speakers, illiterate individuals, pregnant women, and mentally or developmentally disabled individuals. Adults living in certain group quarters were excluded from the survey, such as adults living in college dormitories and military or other noninstitutional group quarters, a population which comprises 3.1% of the total population of New York City (9).

#### Recruitment

NYC HANES participants were recruited through an initial household visit by field staff members and the completion of an eligibility screening questionnaire. Each household identified by the survey team was mailed an introductory letter 1 to 3 weeks before field staff visited the household. The letter described the study, identified the household as being part of the survey sample, and gave information about the field staff who would be visiting the household in the following 2 weeks. Field staff then visited each household and attempted to speak with an adult resident. Adults were eligible to participate in the eligibility screening questionnaire if the household visited was their usual place of residence. Field staff visited households 7 days per week and at varying hours of the day to accommodate varying work schedules. If an adult was present, the field staff member explained the survey and completed a screening questionnaire on household composition, including the name (or initials), age, and sex of each household member. Eligible survey participants were identified and invited to make an appointment at one of the NYC-HANES–dedicated health centers for an interview, a physical examination, and a biologic specimen collection. Survey staff sent a reminder letter 1 week before the appointment date and made a reminder telephone call the evening before the scheduled appointment.

When the potential participants were screened at their households, field staff described the benefits of participation in NYC HANES. Each survey participant received $100 (two $50 postal money orders) upon completion of the survey and laboratory tests valued at more than $300. For survey participants who were elderly, infirm, or otherwise in need of transportation, taxi service was also provided to and from the selected health center. All outreach materials were prepared at a sixth-grade reading level and translated into Spanish, Russian, Chinese, Korean, French Creole, Arabic, and Italian. If selected household members spoke a language not covered by NYC HANES staff, a telephone-based language translation service was used to gain cooperation and to complete the screening form.

### Data collection 

Household screening and survey data collection were conducted between June 2 and December 19, 2004. Data collection for study participants consisted of three components: a two-part interview, a physical examination, and a biologic specimen collection. The average time required to participate in all three components of the survey was 1 hour and 40 minutes.

#### Interview

The interview consisted of two parts: a face-to-face interview and an audio computer-assisted self-interview (CASI). (Copies of survey instruments are available from http://www.nyc.gov/html/doh/html/hanes/hanes.shtml.) In the face-to-face interview, trained interviewers asked participants questions about their health behaviors, medical history, health care access and use, nutrition, and demographic information. The audio CASI used a fully automated computer interface to prompt questions and to record responses in either English or Spanish. All interview materials were prepared for comprehension at a sixth-grade reading level. For participants who spoke another language, either a staff member or family-member proxy translated the interview for the participant. If neither was available, a telephone-based language translation service was used.

The face-to-face interview also included an assessment of mental health status using the Composite International Diagnostic Interview of the World Health Organization (12). These interviews were conducted primarily in English or Spanish. NYC HANES staff or language-translation services were used to translate this component into other languages. Participants who had family members translating for them did not complete this component of the survey for confidentiality reasons.

All participants exhibiting psychological distress were given a brochure on where to obtain free referrals to mental health support services and were given the opportunity to speak with a trained mental health counselor before leaving the health center. The health center coordinator consulted with an on-call psychiatrist to determine an appropriate referral for participants expressing severe emotional distress or current or recent suicide ideation.

The second part of the interview focused on sensitive topics, including drug use, sexual behavior, incarceration, and domestic violence. These questions were asked using audio CASI in English or Spanish. The participant listened to a recorded voice through a headset while reading the questions on the screen and then indicated responses to each question by touching the computer screen; the process provided total privacy for the participant. Because of the sensitivity of the questions, this component was not included in interviews using proxy translators. Randomized controlled trials comparing data from audio CASI with data from face-to-face interviews have shown that sensitive behaviors are more frequently reported in settings that use audio-CASI technologies (13-15).

#### Physical examination

The physical examination was limited to basic anthropometry and blood pressure measurements. Participants were asked to change into disposable gowns to facilitate the measurement process and standardize weight measures. All measurements were made using standardized NHANES protocols and equipment (16).

#### Biologic specimen collection

Blood and urine specimens were collected from all consenting survey participants. Approximately 32 ml of blood were drawn from participants. All specimens were processed according to protocol, and aliquots were shipped to laboratories to be analyzed or stored for future research.

#### Home interview option

To maximize participation, a home interview component was introduced in September 2004 and offered to participants who were elderly, infirm, pregnant, or living in remote neighborhoods and to participants who were too busy or unwilling to schedule a full interview and examination at the NYC-HANES–dedicated health center. The home interview included anthropometric measurements, blood pressure measurements, and the face-to-face interview. The mental health interview, audio CASI, and blood and urine specimen collection were not conducted in the home. At the end of the home interview, participants were encouraged to make an appointment for a brief 20- to 30-minute visit to one of the NYC-HANES–dedicated health centers to complete the survey. Remuneration for the home interview was $50; the remaining $50 was given upon completion of the health center component.

### Data management 

The New York City Department of Health and Mental Hygiene collaborated with and provided funding to the National Center for Health Statistics to develop the NYC HANES data collection applications. The applications, collectively known as the *Community HANES Information Technology Architecture*, were developed in a modular format to ensure flexibility and facilitate adaptation to future HANES initiatives. The architecture was based on the NHANES framework of software, database, and concepts and was designed to ensure data security and immediate access to data. Data were synchronized between the central office of the New York City Department of Health and Mental Hygiene and the NYC-HANES–dedicated health centers.

Upon completion of the survey, an analytic database was developed, stripped of identifying information, and placed on a separate analytic server. All subsequent data analyses used this database to protect the confidentiality of survey participants. Only the study director and data manager retained permission rights to access participant identifiers. Survey data were weighted to adjust for the complex sampling design, nonresponse, and post-stratification. 

### Quality assurance 

Several measures were instituted to ensure the quality of the body measures and blood pressure data. Staff members from the National Center for Health Statistics and a blood pressure consultant trained all health technicians on proper measurement techniques according to the standardized NHANES protocol. Blood pressure training covered several techniques intended to reduce observer bias (17). Health technicians were required to pass tests developed and administered by Shared Care Research and Education & Consulting, Inc (Milwaukee, Wisc) before conducting anthropometry and blood pressure measurements. Of 22 individuals trained as technicians, 18 passed all necessary components. Mid-survey, all technicians were retrained and tested. Only staff members who were successfully recertified (15 of 18) were allowed to continue collecting blood pressure and body measurement data.

Quality assurance reports were prepared and monitored on a weekly basis. Means and distributions for waist circumference, height, weight, systolic blood pressure, and diastolic blood pressure were evaluated for each health technician. Blood pressure data were also reviewed to identify end-digit preference, number of missed readings, and frequency of enhancement use. Health technicians were also directly observed on a semi-weekly basis.

To ensure that the laboratory-reported values were accurate and reliable, standard quality-control procedures were implemented at each of the testing facilities. These procedures included the analysis of control specimens for which known values or concentrations have been previously assigned. Control specimens were included in each analytical run. Results from analytical runs in which the control specimen readings were out of range were not reported. To monitor the reproducibility of results, a 2% random sample of specimens was repeated.

### Notification of findings and referrals 

All survey participants were notified of their examination findings. Indicators of serious health problems were reported as soon as they were detected either during the examination or after laboratory tests or diagnostic readings were performed. For emergent conditions, staff members discussed the findings (either at the clinic or over the telephone) with the examinee and urged the individual to see a medical provider immediately for a complete evaluation. This process included participants expressing active suicide ideation, hypertension, fasting plasma glucose greater than 350 mg/dL, or triglyceride levels of greater than 1000 mg/dL. Participants with fasting plasma glucose levels greater than 125mg/dL with no prior diagnosis of diabetes were also called by a nurse practitioner and encouraged to obtain a full diabetes assessment. Positive test results for hepatitis C and herpes simplex virus type 2 antibodies were provided by letter; participants with a prior diagnosis of diabetes were also informed by letter of elevated diabetes indicators. A final report of all examination findings, including laboratory tests, was sent within 12 to 16 weeks of the examination. A local telephone number was provided to all participants for consultation and referral information.

## Results

A total of 4026 households were approached by survey field staff (Figure). Eligibility screening questionnaires were completed for 3388 (84%) of remaining households. Of the 638 (16%) households for which individuals did not complete questionnaires, refusals accounted for 12%; other questionnaires were not completed because of an inability to enter the home, the illness of a resident, language barriers, or an inability to reach residents. Screening response rates among segments varied between 44% and 100%.

Of the 3388 households with completed eligibility questionnaires, 3047 survey participants were identified. Of these survey participants, 2306 (76%) made an appointment, and 1999 (66%) completed the survey. The overall response rate was 55%.

## Discussion

The NYC HANES survey represents the first community-level attempt to replicate core aspects of NHANES, the cornerstone health examination survey in the United States. The New York City Department of Health and Mental Hygiene, with support from the National Center for Health Statistics, conducted NYC HANES to improve disease surveillance and establish citywide estimates for several previously unmeasured health conditions from which reduction targets could be set and incorporated into health policy planning initiatives (18). NYC HANES will also provide important new information about the prevalence and control of chronic disease precursors, such as undiagnosed hypertension, hypercholesterolemia, or impaired fasting glucose, which will allow chronic disease programs to monitor more proximate health events and rapidly evaluate primary intervention efforts. Study findings will be used by the public health community in New York City, as well as by researchers and clinicians, to better target resources to the health needs of the population.

## Figures and Tables

**Table 1:** Components of the New York City Health and Nutrition Examination Survey, 2004


**Two-part interview**

Face to face
Computer-assisted self-interview

**Physical examination**

Systolic blood pressure
Diastolic blood pressure
Pulse
Standing waist girth
Weight
Height
Arm circumference

**Biologic specimen collection**

**Blood**
Lipid profile
Total cholesterol
High-density lipoprotein
Low-density lipoprotein^a^
Triglycerides^a^
Plasma glucose^b^
Hemoglobin A1c
Hepatitis C virus antibody
Herpes simplex type 1 and type 2
Serum cotinine
Serum heavy metals
Cadmium
Lead
Mercury
**Urine**
Trace metals
Arsenic
Antimony
Barium
Beryllium
Cadmium
Cesium
Cobalt
Lead
Molybdenum
Platinum
Thallium
Tungsten
Uranium
Pesticides
Malathion dicarboxylic acid
* para*-Nitrophenol
3,5,6-Trichloro-2-pyridinol
2-Isopropyl-4-methyl-6-hydroxypyrimidine

a Test was administered only to participants who had fasted for 9 or more hours.

b Test was administered only to participants who had fasted for 8 or more hours.

**Table 2:** Sample Design Parameters for New York City Health and Nutrition Examination Survey, 2004

**No. of Eligible Adults in House-hold**	**No. of Households in New York City**	**Adult Population in New York City**	**No. of Eligible Adults Selected in Household**	**Estimated No. of Households With Completed Eligibility Questionnaires**	**Sampling Rate Used to Designate Households for Sampled Adult Selection^a^**	**Overall Proposed Sampling Rate for Selecting Adults**	**Esti-mated No. of Selected House-holds With Sampled Adults**	**Esti-mated No. of Sampled Adults**
1	1,170,514	1,170,514	1	1101	0.000588	0.0005	550	550
2	1,677,047	3,354,095	1	1577	0.001175	0.0005	1577	1577
3	260,158	780,473	2	245	0.000881	0.0005	183	366
4	66,613	266,451	2	63	0.001175	0.0005	63	126
≥5	20,173	100,865	2	19	0.001175	0.0004	19	38
Group quarters^b^	b	182,430	0	0	0	0	0	0
Total	3,194,505	5,854,828	—	3004	—	—	2392	2657

a Rates were inflated to account for expected screener nonresponse of 20%. During the sample design process, it was found that a reduction in screener nonresponse could be realized by reducing the rate for households with five or more eligible persons to 0.001175 (the maximum among other household sizes), with a negligible loss of statistical efficiency.

b Adults living in certain group quarters were excluded from the survey, such as adults living in college dormitories and military or other noninstitutional group quarters.

## References

[1] Hajjar I, Kotchen TA (2003). Trends in prevalence, awareness, treatment, and control of hypertension in the United States, 1988–2000. JAMA.

[2] Harris MI, Flegal KM, Cowie CC, Eberhardt MS, Goldstein DE, Little RR (1998). Prevalence of diabetes, impaired fasting glucose, and impaired glucose tolerance in U.S. adults. The Third National Health and Nutrition Examination Survey, 1988–1994. Diabetes Care.

[3] Tormo MJ, Navarro C, Chirlaque MD, Barber X (2000). Validation of self diagnosis of high blood pressure in a sample of the Spanish EPIC cohort: overall agreement and predictive values. J Epidemiol Community Health.

[4] (2001). National Center for Health Statistics. National Health and Nutrition Examination Survey: Interviewer procedures manual: March 2001.

[5] (2000). Centers for Disease Control and Prevention. 2000 CDC growth charts for the United States: methods and development. Series report 11.

[6] Butterworth CE, Bendich A (1996). Folic acid and the prevention of birth defects. Annu Rev Nutr.

[7] Pirkle JL, Brody DJ, Gunter EW, Kramer RA, Paschal DC, Flegal KM (1994). The decline in blood lead levels in the United States. The National Health and Nutrition Examination Survey (NHANES). JAMA.

[8] New York City Department of Health and Mental Hygiene. NYC community health data: technical notes.

[9] (2001). United States Census Bureau. Census 2000 summary file 1: New York.

[10] (2001). United States Census Bureau. Current population survey (CPS): March supplement datafile.

[11] Montaquila JM, Mohadjer L, Curtin LR, Gwynn RC, Thorpe L NYC HANES: Design of a community health and nutrition examination survey. Proceedings of the American Statistical Association 2004, Survey Research Methods Section [CD-ROM].

[12] Wittchen HU (1994). Reliability and validity studies of the WHO—Composite International Diagnostic Interview (CIDI): a critical review. J Psychiatr Res.

[13] Williams ML, Freeman RC, Bowen AM, Zhao Z, Elwood WN, Gordon C (2000). A comparison of the reliability of self-reported drug use and sexual behaviors using computer-assisted versus face-to-face interviewing. AIDS Educ Prev.

[14] Krawczyk CS, Gardner LI, Wang J, Sadek R, Loughlin AM, Anderson-Mahoney P (2003). Test-retest reliability of a complex human immunodeficiency virus research questionnaire administered by an Audio Computer-assisted Self-interviewing system. Med Care.

[15] Metzger DS, Koblin B, Turner C, Navaline H, Valenti F, Holte S (2000). Randomized controlled trial of audio computer-assisted self-interviewing: utility and acceptability in longitudinal studies. HIVNET Vaccine Preparedness Study Protocol Team. Am J Epidemiol.

[16] Centers for Disease Control and Prevention. National Health and Nutrition Examination Survey (NHANES) questionnaire and exam protocol [Internet].

[17] Ostchega Y, Prineas RJ, Paulose-Ram R, Grim CM, Willard G, Collins D (2003). National Health and Nutrition Examination Survey 1999–2000: effect of observer training and protocol standardization on reducing blood pressure measurement error. J Clin Epidemiol.

[18] Henning KJ, Bassett MT, Silver L, Sederer LI, Lyman A, Frieden TR (2004). Take care New York: a policy for a healthier New York City. City Health Information.

